# The *Burkholderia pseudomallei* Type III Secretion System and BopA Are Required for Evasion of LC3-Associated Phagocytosis

**DOI:** 10.1371/journal.pone.0017852

**Published:** 2011-03-11

**Authors:** Lan Gong, Meabh Cullinane, Puthayalai Treerat, Georg Ramm, Mark Prescott, Ben Adler, John D. Boyce, Rodney J. Devenish

**Affiliations:** 1 Department of Biochemistry and Molecular Biology, Monash University, Melbourne, Victoria, Australia; 2 Department of Microbiology, Monash University, Melbourne, Victoria, Australia; 3 Australian Research Council Centre of Excellence in Structural and Functional Microbial Genomics, Monash University, Melbourne, Victoria, Australia; Indian Institute of Science, India

## Abstract

*Burkholderia pseudomallei* is the causative agent of melioidosis, a fatal infectious disease endemic in tropical regions worldwide, and especially prevalent in southeast Asia and northern Australia. This intracellular pathogen can escape from phagosomes into the host cytoplasm, where it replicates and infects adjacent cells. We previously demonstrated that, in response to *B. pseudomallei* infection of macrophage cell line RAW 264.7, a subset of bacteria co-localized with the autophagy marker protein, microtubule-associated protein light chain 3 (LC3), implicating autophagy in host cell defence against infection. Recent reports have suggested that LC3 can be recruited to both phagosomes and autophagosomes, thereby raising questions regarding the identity of the LC3-positive compartments in which invading bacteria reside and the mechanism of the autophagic response to *B. pseudomallei* infection. Electron microscopy analysis of infected cells demonstrated that the invading bacteria were either free in the cytosol, or sequestered in single-membrane phagosomes rather than double-membrane autophagosomes, suggesting that LC3 is recruited to *B. pseudomallei*-containing phagosomes. Partial or complete loss of function of type III secretion system cluster 3 (TTSS3) in mutants lacking the BopA (effector) or BipD (translocator) proteins respectively, resulted in delayed or no escape from phagosomes. Consistent with these observations, *bopA* and *bipD* mutants both showed a higher level of co-localization with LC3 and the lysosomal marker LAMP1, and impaired survival in RAW264.7 cells, suggesting enhanced killing in phagolysosomes. We conclude that LC3 recruitment to phagosomes stimulates killing of *B. pseudomallei* trapped in phagosomes. Furthermore, BopA plays an important role in efficient escape of *B. pseudomallei* from phagosomes.

## Introduction

*Burkholderia pseudomallei* is a Gram-negative, soil dwelling bacillus. It is the causative agent of melioidosis, a fatal infection of many animal species and humans and is endemic in tropical and subtropical areas of the world [1], [2]. Melioidosis generally presents as a febrile illness ranging from acute pneumonia or septicemia to chronic abscesses; prolonged periods of latency have also been documented [3]. The overall mortality associated with melioidosis remains high; at approximately 40% in northeast Thailand and 20% in northern Australia [2], [4]. While some *B. pseudomallei* virulence factors have been identified including capsule, flagella, lipopolysaccharide (LPS), pili, quorum sensing molecules and the type III secretion system cluster 3 (TTSS3), our current understanding of *B. pseudomallei* pathogenesis remains incomplete (reviewed in [1], [5], [6]).

*B. pseudomallei* is an intracellular pathogen that can invade both phagocytic [7] and non-phagocytic cells [8]. Following internalization, bacteria can escape from the phagosome into the host cytoplasm in a process that is dependent on a functional TTSS3 [9]. Once in the cytoplasm bacteria can replicate and induce actin polymerization at one pole of the bacterium, facilitating intracellular motility [10]. This actin-based motility is considered to facilitate bacterial spreading into adjacent cells leading to the formation of multinucleated giant cells (MNGC), which have been observed both in cultured cell lines and the tissues of patients [11].

Autophagy is a cellular degradation system that eliminates unwanted molecules, damaged proteins and organelles from within the cell and it plays an important role in many physiological and pathological processes, including the cellular response to starvation, cell development and tumor suppression (reviewed in [12], [13]). Autophagy is also a component of innate immune defence, as it is involved in the clearance of a variety of pathogenic bacteria [14], [15], [16]. Autophagy is critical for the elimination of cytoplasmic Group A *Streptococcus* [17] and inhibits the intracellular survival of *Mycobacterium tuberculosis* [18]. However, some host-adapted intracellular pathogens including *Shigella flexneri*, *Listeria monocytogenes* and *Salmonella enterica* serovar Typhimurium, have developed means to evade killing by autophagy. The molecular strategies used by some pathogens to evade autophagy have been reported [19], [20], [21], [22]. Other pathogens can divert phagosome maturation towards the autophagy pathway, taking control of this host defence pathway to the benefit of the pathogen [23], [24]. *Legionella pneumophia*, *Coxiella burnetii*, *Brucella abortus*, *Porphyromonas gingivalis*, *Staphylococcus aureus*, and *Anaplasma phagocytophilum* each exploit modified autophagosomes as their intracellular niche [25], [26], [27], [28], [29].

We have shown that, in response to *B. pseudomallei* infection of macrophage cell line RAW 264.7, only a subset of bacteria co-localized with the autophagy marker protein LC3 [30]. When cells were treated with rapamycin, an inducer of autophagy, bacterial co-localization with LC3 was significantly increased and bacterial survival reduced. Thus, autophagy was implicated as part of the host defence system against *B. pseudomallei* infection, although the strategy by which most invading bacteria avoided host autophagic attack remained unclear. Moreover, we showed the involvement of the bacterial TTSS3-effector protein BopA in modulating the host cell response, as *bopA* mutant bacteria showed increased co-localization with LC3 and reduced intracellular survival [30].

A recent report showed that LC3 can be recruited directly to bacteria-containing phagosomes [31]; a process designated LC3-associated phagocytosis (LAP) [32]. In RAW 264.7 cells infected with *Escherichia coli*, LAP was induced by lipopolysaccharide (LPS) via toll-like receptors (TLR), and involved the rapid translocation of the autophagic proteins Beclin1 and LC3 to the bacteria-containing phagosomes, leading to an increased level of phagocytosis and bacterial killing [31]. Such reports led us to assess the nature of the compartment in which intracellular *B. pseudomallei* is sequestered. Here we demonstrate, through analysis of electron microscopic (EM) images of infected cells, that intracellular *B. pseudomallei* bacteria are free in the cytosol or sequestered in single-membrane phagosomes, but rarely in double-membrane autophagosomes, suggesting that LC3 is recruited to *B. pseudomallei*-containing phagosomes. Furthermore analysis of a mutant lacking a functional TTSS3 showed that when bacteria are unable to escape from the phagosome they become strongly co-localised with LC3, confirming that LC3 is recruited to *B. pseudomallei*-containing phagosomes. A high percentage of LC3 positive phagosomes become LAMP1 positive, implicating LC3 recruitment in efficient phagosome maturation. Importantly, we also show that efficient escape of *B. pseudomallei* from phagosomes requires the presence of the predicted TTSS3 effector protein BopA. Finally, as we were unable to observe more than a single bacterium within a double membrane vesicle, we conclude that *B. pseudomallei* can efficiently avoid engulfment by canonical autophagosomes. Taken together these data show that LC3 recruitment to *B. pseudomallei*-containing phagosomes plays a role in destruction of bacteria trapped in phagosomes, but that most bacteria can escape from the phagosome and once free in the cytoplasm are rarely targeted by autophagosomes.

## Materials and Methods

### Bacterial strains and cell culture

*B. pseudomallei* wild-type strain K96243 [33] and mutants were cultured in Luria–Bertani (LB) broth at 37°C. *E. coli* strain SM10*λpir* was used as a conjugative donor of the *λpir*-dependent suicide replicon pDM4 (*ori* R6K, *mob* RP4, *sacBR*, *cat*) and its derivatives [34]. The RAW 264.7 cell line stably expressing GFP-LC3 was constructed as described [30]. Cells were maintained at 37°C in 5% CO_2_ without antibiotics in RPMI 1640 medium (Gibco Laboratories), supplemented with 10% (v/v) heat-inactivated fetal bovine serum (JRH Biosciences). Bacteria were heat-killed at 98°C for 30 min. The rat anti-LAMP1 antibody was obtained from the Development Studies Hybridoma Bank developed by J.T. August under the auspices of the NICHD and maintained by The University of Iowa, Department of Biological Sciences, Iowa City, IA. All other chemicals were purchased from Sigma unless otherwise indicated.

### Mutagenesis of TTSS3 genes

**Figure 1:**
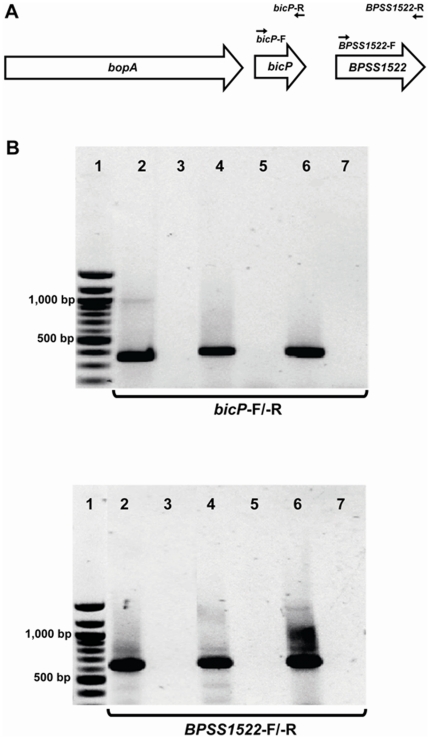
Reverse transcription (RT)-PCR of *bicP* and *bpss1522* in wild-type and *bopA* mutant *B. pseudomallei*. RNA extracted from the wild-type K96243 or *bopA* deletion mutant strain was reverse-transcribed to obtain cDNA. Each cDNA preparation was used as a template for RT-PCR. (A) Schematic diagram showing the position of primers used for RT-PCR. (B) Electrophoretic separation of RT-PCR products amplified with primers for *bicP* (top panel) and *bpss1522* (lower panel). These products were generated from reactions: (2) *bopA* mutant cDNA; (3) *bopA* mutant cDNA, no RT control; (4) wild-type cDNA, (5) wild-type cDNA, no RT control; (6) wild-type genomic DNA control; and (7) No DNA control. DNA size markers (bp) are shown in lane 1.

The *B. pseudomallei bopA* deletion mutant has been described previously [30]. This mutant is unmarked and contains an in-frame deletion within *bopA*. Although we were unable to complement this mutant, we have subsequently used RT-PCR to analyse the transcription of the *bicP* and *bpss1522* genes, which are downstream of *bopA*. Primer pairs (bicP-F, 5′-AACGTGTCGATCAGGCTTTC-3′ and bicP-R, 5′-ACGCACACCGAATGGTTGAA-3′) and (BPSS1522-F, 5′-GGCGCGCACGCGTTCGCATA-3′ and BPSS1522-R, 5′- GGGTGCTCGTCGTCGACAGC-3′) were used to amplify *bicP* and *bpss1522* RT-PCR products respectively. RT-PCR products of the expected size, 368 bp (*bicP*) and 637 bp (*bpss1522*) were generated from cDNA derived from both the wild-type and *bopA* mutant strains (Figure 1), indicating that the deletion mutation within *bopA* does not disrupt the transcription of these genes.

A *B. pseudomallei bapA* mutant was constructed by double-crossover allelic exchange using the λ*pir*-dependent vector pDM4 which carries the negative-selectable marker *sacB* [34]. PCR primer pairs (5′-GGGCCCACTAGTCCGATCCGAAGCAACCGACAAGA-3′ and 5′-GGGCCCAGATCTACCATGTCGACGAGATTCGTC-3′; 5′-GGGCCCAGATCTCTTTATCCGCTCGTCGACGATGCTT-3′ and 5′-GGGCCCTCTAGATTGGCGTATTGGCGTATTGGCGTA-3′) were used to amplify upstream and downstream sequences flanking the *bapA* open reading frame, respectively. A 918-bp fragment spanning the 5′ section of *bapA* and upstream DNA was cloned into *Spe*I/*Bgl*II digested pBluescript (Stratagene) then a 1,241 bp downstream fragment spanning the 3′ section of *bapA* and downstream DNA was cloned into the *Bgl*II/*Xba*I sites. The tetracycline resistance cassette *tetA*(C) was digested from the plasmid pUTminiTn*5* [35] and ligated into the *Bgl*II site between the two cloned *bapA* fragments, generating a plasmid containing a 1,565 bp internal deletion in *bapA*. The mutagenesis construct was then transferred to pDM4. This pDM4 derivative was introduced into *B. pseudomallei* by conjugation and transconjugants selected on plates containing tetracycline (25 µg/ml). Colonies were then screened for chloramphenicol sensitivity in the presence of sucrose.

A *bipD* mutant was constructed by single crossover insertional mutagenesis. PCR primers (5′-GGGCCCACTAGTAACCTGCTCGAGCGCCTGGAAA-3′ and 5′-GGGCCCTCTAGAGCCGCCGTCGATCTTCATGT-3′) were used to amplify a 261 bp internal fragment from within the target open reading frame. This fragment was digested with the appropriate enzymes, ligated to *Spe*I/*Xba*I-digested pDM4 and introduced into *B. pseudomallei* as above. Single crossover mutants were selected on LB plates containing chloramphenicol (50 µg/ml). Each of the mutants was verified by PCR and sequence analysis.

### Bacterial replication assays

To determine the ability of *B. pseudomallei* strains to replicate intracellularly, RAW 264.7 cells stably expressing green fluorescent protein-LC3 (GFP-LC3) were infected with *B. pseudomallei* K96243 as previously described [30]. Briefly, RAW 264.7 cells (seeded at 1.0×10^5^ cells/well) in 24-well trays (BD Biosciences) were infected with *B. pseudomallei* at a multiplicity of infection (MOI) of 10∶1, and incubated at 37°C for 1 h to allow bacterial invasion. Infected cells were washed 3 times with phosphate buffered saline pH 7.2 (PBS) and then inoculated into fresh medium supplemented with kanamycin (800 µg/ml) and ceftazadime (800 µg/ml) to kill extracellular bacteria. At 2, 4 and 6 h after addition of bacteria, cells were washed 4 times with PBS and intracellular bacteria were released by addition of 0.1% Triton X-100. For each time point, cell lysates were prepared from cells grown in 3 separate wells. Serial dilutions of each lysate were plated onto LB agar and the numbers of intracellular bacteria (expressed as colony forming units (CFU)), were enumerated by direct colony counts after 48 h. Bacterial survival was normalised to counts obtained at 2 h post-infection (p.i.), the time point at which extracellular bacteria were killed with antibiotics, and data presented as relative survival (%).

### Immunofluorescence and confocal microscopy

To determine co-localization with GFP-LC3, cells were cultured in 24-well trays (BD Biosciences) containing 13-mm-diameter glass coverslips (ProScitech). At the indicated time points p.i., the cells were fixed with methanol for 10 min and blocked for 1 h in PBS containing 1% (w/v) bovine serum albumin (BSA) and 0.1% (v/v) Triton X-100. Coverslips were incubated with rabbit anti-*B. pseudomallei* outer membrane serum [30] at a 1∶100 dilution. The fluorescently labeled secondary antibody Alexa Fluor 405-conjugated goat anti-rabbit IgG serum (Molecular Probes) was used at a 1∶250 dilution. LAMP1 was labeled with rat anti-LAMP1 antibody (Development Studies Hybridoma Bank, University of Iowa, IA) at a 1∶100 dilution followed by Alexa Fluor 568-conjugated goat anti-rat IgG serum (Molecular Probes) at a 1∶250 dilution. Stained cells were washed with PBS, coverslips mounted in Permafluor aqueous mounting medium (Immunotech) and visualised using a confocal laser scanning microscope (CLSM; Olympus FV500) equipped with a 1.2 NA water immersion lens (Olympus 60X UPlanapo). Image analysis and processing was performed using Olympus FluoView TIEMPO software version 4.3 and the public domain software Image-J version 1.41a (http://rsb.info.nih.gov/ij). The intracellular location of bacteria was assessed using Z-stack CLSM analysis. For actin staining, RAW 264.7 cells, cultured on coverslips at the indicated time points p.i., were fixed in 3.5% (w/v) para-formaldehyde (PFA) and 0.1% (v/v) Triton X-100 consecutively for 10 min prior to staining as described above. Stained cells were counter stained with Alexa-Flour 488 conjugated phalloidin (Molecular Probes) at a 1∶500 dilution for 50 min to visualize cellular actin filaments.

### Transmission electron microscopy

For standard transmission electron microscopy, infected (at a MOI of 10 or 100) and uninfected RAW 264.7 cells were fixed for 2 h at room temperature with 2.5% (w/v) glutaraldehyde (EM Sciences) in 0.1 M cacodylate buffer, pH 7.2 at the indicated time points. Cells were collected, washed twice and postfixed for 1 h at room temperature with 1% (w/v) osmium tetroxide and then subsequently incubated with 2% (w/v) uranyl acetate for 1 h. After dehydration and embedding in Epon resin (EM Sciences), ultra-thin 70 nm sections were cut and stained with lead citrate and uranyl acetate. Sections were viewed at 80 kV using a Hitachi H-7500 transmission electron microscope fitted with a Gatan Multiscan 791 CCD camera.

### Statistical analyses

For quantification studies, at least 100 bacteria were counted for each condition in each experiment, unless otherwise indicated. Values were expressed as mean ± standard error of the mean (SEM). Statistical analysis was performed using GraphPad Prism software version 5.00 (http://www.graphpad.com). Differences between groups were analyzed by 2-tailed, 2-sample, unequal variance Student's t test or ANOVA analysis with Dunn post-hoc test where appropriate. A *p* value of <0.05 was considered to be statistically significant.

## Results

### Intracellular *B. pseudomallei* are localized free in the cytosol or in phagosomes

**Figure 2:**
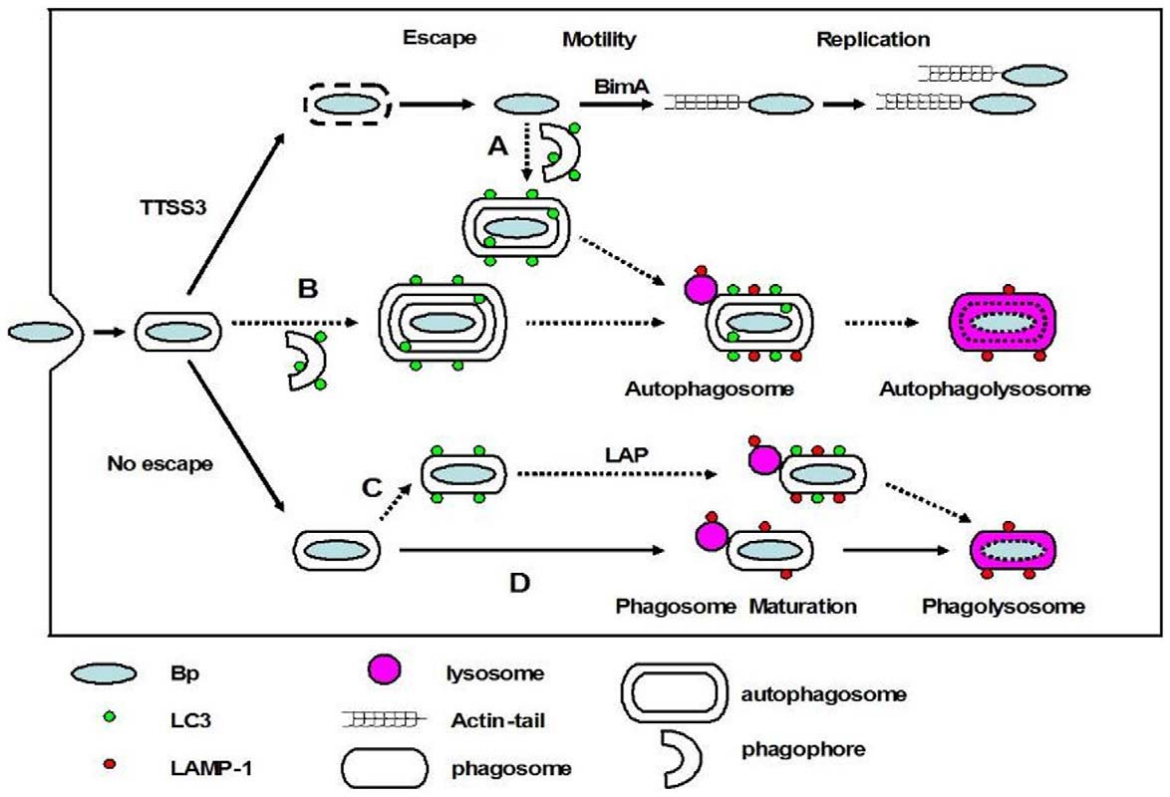
Possible fates of *B. pseudomallei* in infected macrophages. Following phagocytic uptake by macrophages, bacteria are first located within phagosomes. The majority of wild-type *B. pseudomallei* (Bp) can escape from the phagosome into the cytosol in a process which is largely uncharacterized but involves the TTSS3. Once free in the cytosol bacteria activate BimA-mediated actin-based motility, replicate and invade adjacent cells via membrane protrusions. Potentially some cytosolic bacteria may be sequestered in canonical autophagosomes (pathway A). Some bacteria which remain in phagosomes might be sequestered by double-membrane autophagosomes (pathway B). The autophagy marker protein LC3 can be recruited to bacteria-containing phagosomes, a process designated LC3-associated phagocytosis (LAP) which stimulates further phagosomal maturation via recruitment of other proteins including LAMP1, a late endosome/lysosome marker, and the subsequent fusion of phagosomes with lysosomes, leading to bacterial killing (pathway C). Finally phagosomes may mature to phagolysosomes without LC3 recruitment (pathway D).

**Figure 3:**
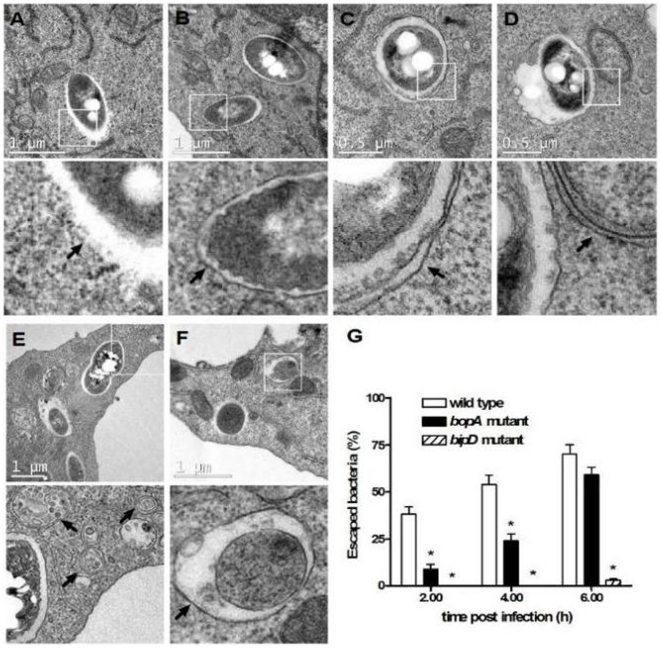
*B. pseudomallei*-containing vacuoles are bound by single membranes and the TTSS3 and the effector BopA are required for bacterial escape. (A–F) Transmission electron micrographs show the intracellular location of *B. pseudomallei* in RAW 264.7 cells at 2–6 h post infection (p.i.). The scale bar is indicated. Boxed areas are shown as magnified images below each panel. Intracellular bacteria were observed either free in the cytosol and not membrane bound (panel A), or within single-membrane phagosomal compartments (panel B). Only one bacterium was found in a double-membrane compartment (panel C), which could be an autophagosome. Canonical autophagosomes having a double-membrane were observed in infected and uninfected RAW 264.7 cells (panels D–F). Arrows indicate detailed membrane ultrastructure. (G) The percentage of bacteria free in the cytosol of RAW 264.7 cells following infection with *B. pseudomallei* wild-type, *bopA* mutant and the *bipD* mutant at 2, 4, and 6 h p.i. Data represent the mean ± SEM of three separate experiments (n = 100 bacteria). Where shown * indicates p<0.05 relative to the wild-type strain at each time point.

Only a small subset of intracellular *B. pseudomallei* co-localizes with the autophagy marker protein LC3 in infected RAW 264.7 macrophage cells, although this co-localization increases when cells are treated with the autophagy inducer rapamycin [30]. However, intracellular pathogens may be subject to LC3-dependent host autophagic processes by three distinct pathways as depicted in Figure 2: A) autophagosomes may directly sequester bacteria free in the cytosol [21], [36]; B) autophagosomes may engulf bacteria-containing phagosomes [18]; C) LC3 may be recruited directly to bacteria-containing phagosomes thus stimulating phagosome maturation [31]. The operation of each of these pathways could give rise to co-localization of bacteria with LC3. To investigate which of these pathways may be responsible for co-localization of *B. pseudomallei* with LC3 in infected RAW 264.7 cells, we used transmission electron microscopy (TEM) to view sections prepared from cells infected with wild-type bacteria (at MOI of 100) (Figure 3).

Cell sections were scored for the presence of bacteria and whether the bacteria were within single-membrane compartments (phagosomes), double- or multi- membrane compartments (autophagosomes) or unbounded by any detectable membranes (free in the cytosol). These analyses showed that *B. pseudomallei* was primarily present either free in the cytoplasm (Figure 3A), or within single-membrane phagosomes (Figure 3B). For wild-type *B. pseudomallei* the proportion of bacteria free in the cytosol was 39%, 54% and 71% at 2, 4 and 6 h p.i. respectively (Figure 3G), while the proportion of bacteria within phagosomes was 61%, 46% and 29% at 2, 4 and 6 h p.i. respectively. Of the 500 bacteria identified and scored in TEM sections, only a single bacterium was observed within a double-membrane compartment that could be considered to be an autophagosome (Figure 3C). Importantly, the number of canonical double-membrane structures representing different stages of autophagosome maturation (phagophore, autophagosome, amphisome) increased in infected RAW 264.7 cells (Figure 3D–F), confirming our previous observation that autophagy is induced in response to *B. pseudomallei* infection [30]. However, these autophagosome structures did not contain bacteria. Therefore, *B. pseudomallei* is not efficiently targeted by canonical double-membrane autophagosomes via the pathways A or B as shown in Figure 2. However, as we have previously shown that approximately 5–10% of wild-type *B. pseudomallei* are associated with LC3 at 2 h p.i. [30], these results suggest that LC3 may be recruited to *B. pseudomallei*-containing phagosomes (as depicted in pathway C of Figure 2). Qualitatively identical data were obtained by TEM analysis of RAW 264.7 cells infected with wild-type *B. pseudomallei* at MOI of 10.

### The putative TTSS3 effector BopA is required for efficient escape of *B. pseudomallei* from phagosomes

**Figure 4:**
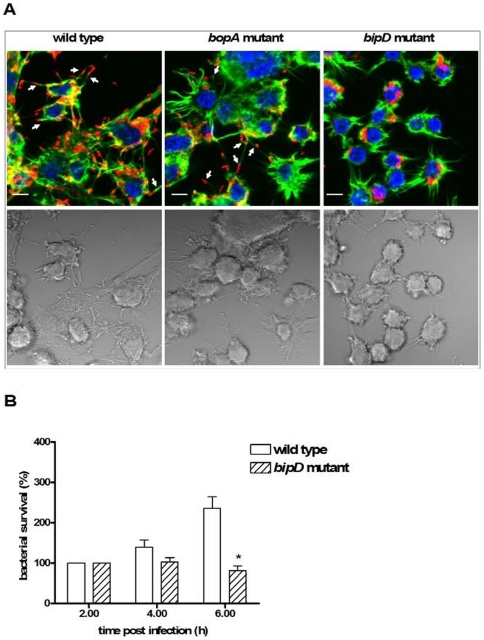
*B. pseudomallei*-associated actin-tails, host cell membrane protrusions and MNGC formation require TTSS3 translocator BipD, but not effector BopA. (A) Representative confocal micrographs with DIC images of RAW 264.7 cells infected with the wild-type strain, the *bopA* mutant, or the *bipD* mutant at 6 h p.i. Bacteria were stained red, filamentous actin was stained green and nuclei were stained blue. Bacteria with associated actin-tails are marked with arrows. Scale bar = 5 µm. (B) Intracellular survival of *B. pseudomallei* wild-type and *bipD* mutant in RAW 264.7 cells at 2, 4, and 6 h p.i. Bacterial survival was normalized to CFU counts obtained at 2 h p.i. and presented as relative survival (%). Data represent the mean ± SEM of three separate experiments, each carried out in triplicate. Where shown * indicates p<0.05 relative to the wild-type strain at each time point.

We next used TEM to analyse the intracellular location of the *B. pseudomallei* TTSS3 *bipD* and *bopA* mutants in RAW 264.7 macrophage cells. BipD is a component of the needle tip complex of the *B. pseudomallei* TTSS3 apparatus [37]; a *bipD* mutant is unable to escape from the phagosomes of macrophage cells up to 6 h p.i. [9]. BopA is a predicted secreted TTSS3 effector protein [38]. As expected the *bipD* mutant was observed only in single membrane phagosomes at 2 and 4 h p.i. and even at 6 h p.i. 98% of bacteria were still localised in phagosomes while the remaining 2% of bacteria were observed free in the cytoplasm. Furthermore, fluorescence microscopy of infected cells labelled with Alexa Fluor 488-conjugated phalloidin showed that the *bipD* mutant did not form actin tails or MNGC (Figure 4A), consistent with the earlier observations of Stevens *et al.* [9], These data confirm a critical role of the TTSS3 in phagosome escape. The *bopA* mutant also showed significantly reduced phagosome escape compared to the wild-type strain with 9% of the *bopA* mutant cells observed free in the cytosol at 2 h p.i., compared to 39% for the wild-type strain. Furthermore, the *bopA* mutant also showed reduced escape at 4 h p.i. (24% compared to 54% for the wild-type strain) and 6 h p.i. (59% compared to 71% for the wild-type strain). Fluorescence microscopy analysis indicated that the *bopA* mutant could form actin tails when free in the cytoplasm and caused MNGC formation in RAW 264.7 cells (Figure 4A), but at later time points compared to wild-type cells (data not shown). Therefore, the predicted TTSS3 effector protein BopA is important for efficient escape from phagosomes.

### *B. pseudomallei* mutants defective in phagosome escape show increased LC3 co-localization

**Figure 5:**
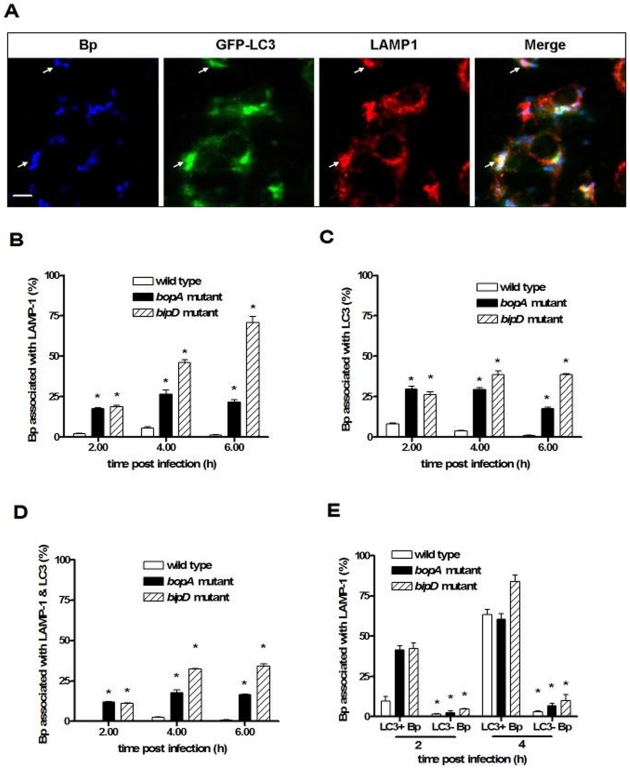
*B. pseudomallei* with defective TTSS3 show enhanced co-localization with autophagy marker protein LC3 and the lysosome marker LAMP1. (A) Confocal images of RAW 264.7 cells expressing GFP-LC3 (green) and infected with *B. pseudomallei* (Bp). Cells were fixed at 2 h p.i., permeabilized, and stained for *B. pseudomallei* (blue) and LAMP1 (red). Arrows indicate bacteria associated with LC3 that are also within LAMP1-positive vacuoles. Bacterial co-localization with LC3 or LAMP1 was defined by the presence of labelled *B. pseudomallei* (blue) which were fully overlaid by intense green/red or fully contained within a green/red ring. Scale bar = 5 µm. (B–D) Quantitative analysis of bacterial co-localization with LC3 (B), LAMP1 (C) or both LC3 and LAMP1 (D) in RAW 264.7 cells infected with *B. pseudomallei* wild-type, *bopA* mutant or *bipD* mutant at 2, 4, and 6 h p.i. (E) The percentage of LC3-positive (LC3^+^ Bp) or LC3-negative bacteria (LC3^−^ Bp) of the wild-type, *bopA* mutant or *bipD* mutant at 2 or 4 h p.i. that co-localized with LAMP1. Data represent the mean ± SEM of three separate experiments (n = 100 bacteria). Where shown * indicates p<0.05 relative to wild-type strain at each time point.

A *B. pseudomallei bopA* mutant displays increased co-localization with LC3 (Figure 5C and [30]). Our TEM data presented here indicate that wild-type *B. pseudomallei* and the *bopA* and *bipD* mutants are very rarely found within double membrane autophagosomes and that the *bopA* mutant displays reduced phagosome escape. Taken together these observations support the proposal that *B. pseudomallei* co-localization with LC3 occurs as a result of recruitment of LC3 to phagosomes that contain bacteria. To further explore this proposal we analysed the co-localization of the *B. pseudomallei bipD* mutant with LC3. Fluorescence microscopy analysis showed that the *bipD* mutant displayed a high level of co-localization with LC3 at 2 h, 4 h and 6 h p.i. (Figure 5C). As shown above, the *bipD* mutant displayed no escape from phagosomes at 2 h and 4 h p.i (Figure 3G). Therefore, this association with LC3 at these time points must result from LC3 recruitment to phagosomes. To investigate whether LC3 recruitment to phagosomes was affected by other TTSS3 effectors, we utilised a mutant defective in another putative effector, BapA, constructed for another study. The *bapA* deletion mutant did not show increased co-localization with LC3 and its ability to escape from phagosomes was indistinguishable from that of wild-type bacteria (data not shown). Collectively these data strongly suggest that all *B. pseudomallei* co-localization with LC3 results from LC3 recruitment to *B. pseudomallei*-containing phagosomes and not to engulfment of cytosolic bacteria by autophagosomes. Thus, the previously reported increased co-localization of a *B. pseudomallei bopA* mutant with LC3 [30] results from the reduced ability of this mutant to escape from phagosomes.

### LC3 recruitment stimulates phagosome maturation

In the process of LC3-associated phagocytosis (LAP), recruitment of LC3 to the phagosome was found to lead to rapid acidification of the compartment and enhanced killing of ingested *E. coli* in RAW 264.7 cells [31], [32]. In an earlier study we demonstrated decreased intracellular survival of the *bopA* mutant [30]. In this study the *bipD* mutant also showed reduced intracellular survival at 6 h p.i. (Figure 4B). Furthermore, *B. pseudomallei* mutants lacking either BipD or BopA display reduced virulence in a mouse melioidosis model [38]. To test the hypothesis that decreased intracellular survival of these mutant bacteria is associated with LAP, we investigated the co-localization of *B. pseudomallei* with LC3 and the lysosome marker lysosomal-associated membrane glycoprotein-1 (LAMP1) in RAW 264.7 cells (Figure 5). LAMP1 is an abundant lysosomal membrane protein that is both delivered to phagosomes during their maturation and required for fusion of lysosomes with phagosomes [39] and autophagosomes [40]. Both the *bopA* and *bipD* mutants showed increased levels of co-localization with LAMP1 (Figure 5B) and LC3 (Figure 5C) compared to the wild-type strain at all time points. At 4 h and 6 h p.i. the *bipD* mutant showed higher levels of co-localization with each marker protein than did the *bopA* mutant. We also determined dual co-localization of bacteria with LC3 and LAMP1 (Figure 5D), as a measure of the maturation of bacteria-containing LC3 phagosomes by fusion with lysosomes. The percentage of bacteria co-localized with both LC3 and LAMP1 was also significantly higher for both the *bopA* and *bipD* mutants (Figure 5D). Importantly, for the wild-type and mutant strains a high percentage of LC3-positive bacteria-containing phagosomes was also LAMP1 positive (Figure 5E), suggesting that LC3 recruitment is associated with enhanced levels of phagolysosome maturation. Consistent with this proposal is the observation that only a very low percentage of LC3-negative bacteria-containing phagosomes are LAMP1 positive but more than 60% of LC3-positive bacteria-containing phagosomes at 4 h p.i. were LAMP1 positive. These data are consistent with the hypothesis that LC3 recruitment to *B. pseudomallei*-containing phagosomes stimulates fusion of phagosomes with lysosomes, leading to enhanced killing of the bacterial contents.

### The recruitment of LC3 to phagosomes is diminished in RAW 264.7 cells which have taken up heat-killed *B. pseudomallei*

**Figure 6:**
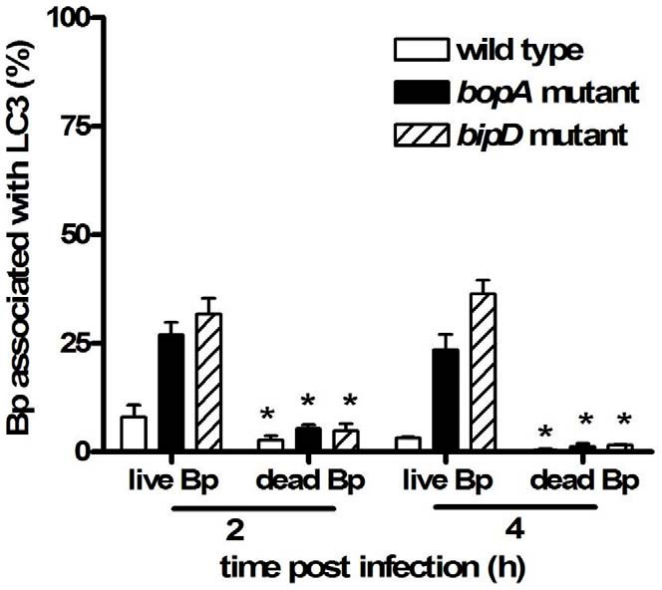
The recruitment of LC3 to bacteria-containing phagosomes is diminished in RAW 264.7 cells infected with heat-killed *B. pseudomallei*. Quantitative analysis of bacterial co-localization with LC3 in RAW 264.7 cells infected with live *B. pseudomallei* (wild-type strain, the *bopA* mutant or the *bipD* mutant) or heat-killed strains at 2 and 4 h p.i. The data represent the mean ± SEM of three separate experiments (n = 100 bacteria). Where shown * indicates p<0.05 relative to the wild-type strain at each time point.

In order to determine whether LC3 recruitment to phagosomes always occurs when *B. pseudomallei* is present within phagosomes, we analysed the co-localization of LC3 with heat-killed bacteria taken up by RAW 264.7 cells. In these experiments only a very small number of internalised bacteria were observed to co-localize with LC3 (Figure 6). Therefore, retention of bacteria within phagosomes is not sufficient for LAP to occur. A low level of co-localization with LC3 was observed for all three heat-killed strains tested: wild-type, *bopA* mutant and *bipD* mutant. Similar results were observed in cells infected with *B. pseudomallei* which had been killed by treatment with PFA or methanol (data not shown). These data suggest that LAP requires the presence of specific bacterial factor(s) produced by viable *B. pseudomallei*.

## Discussion

### LC3-associated phagocytosis (LAP)

TEM analysis of infected RAW 264.7 macrophage cells revealed that invading *B. pseudomallei* bacteria are found either free in the cytosol or sequestered in single-membrane phagosomes (Figure 3). Of 500 bacteria identified and scored in TEM sections only one bacterium was observed within a double-membrane structure likely to be a canonical autophagosome. As we have shown in a previous study that *B. pseudomallei* can be observed co-localized with the autophagosome marker LC3 these data suggest that LC3 is recruited to *B. pseudomallei*-containing phagosomes and that cytoplasmic bacteria are very rarely engulfed by autophagosomes. Moreover, *B. pseudomallei*-containing phagosomes are apparently not targeted by autophagy (Figure 2, pathway B) as no triple membrane structures were observed. These data were supported by analysis of the *B. pseudomallei bipD* mutant which is unable to escape from the phagosome but which showed high levels of co-localization with LC3. Although conventional autophagosomes appear to be rarely involved in clearance of *B. pseudomallei*, LC3-associated phagocytosis (LAP) can be considered to be an autophagy-related mechanism as it requires autophagic proteins Beclin1, Atg5 and Atg7, and is inhibited by the PI3K inhibitors wortmannin or LY294002 [31], [32]. Indeed, treatment of RAW 264.7 macrophage cells with wortmannin reduced the co-localization of the *bopA* mutant with LC3 and increased bacterial survival compared to untreated cells [30].

The LPS of *B. pseudomallei* stimulates both TLR2 and TLR4 while only TLR2 contributes to host defense [41]. We have observed that the treatment of RAW 264.7 cells with *B. pseudomallei* LPS induces a significant increase in the formation of GFP-LC3 puncta [30]. Therefore, in light of the data presented here we propose that LAP in *B. pseudomallei*-infected macrophages is induced in response to LPS, probably via a TLR2- or TLR4-dependent mechanism. Consistent with this proposal TLRs are known to activate the NOX2 NADPH oxidase, and recently it was shown that NOX2-generated reactive oxygen species are necessary for LC3 recruitment to phagosomes [42]. Interestingly we observed that retention of bacteria within phagosomes is insufficient for LAP to occur, as heat-killed *B. pseudomallei* showed dramatically reduced co-localization with LC3 suggesting that LAP requires other bacterial factor(s) produced by, or present on, live bacteria.

### Role of T3SS3 and BopA in the evasion of LAP

The TEM data presented here show the importance of the TTSS3 for escape from phagosomes and identify the putative effector protein BopA as having an important role in phagosome escape. Importantly, bacterial escape from the phagosome results in reduced bacterial killing through avoidance of sequestration within phagolysosomes. The *bipD* mutant showed almost no escape from phagosomes within RAW 264.7 cells up to 6 h p.i.; similar results have been observed for a *B. pseudomallei bipD* mutant within J774.2 murine macrophage-like cells [9]. BipD is predicted to be a component of the secretion needle tip [37], [43] and thus a *bipD* mutant would be unable to secrete any effector proteins. The mechanism by which TTSS3 facilitates phagosome escape is not currently known. Several other putative effector proteins are specified by the *B. pseudomallei* TTSS3 gene cluster [9]. Inactivation of single TTSS3 effector proteins can lead to different outcomes with respect to co-localization with LC3 which presumably reflects the ability of single gene mutants to escape from the phagosome. Thus, the *bopA* mutant exhibited increased co-localization with LC3 and delayed escape from phagosomes, but a mutant defective in another putative effector, BapA, showed a similar level of co-localization with LC3 as the wild-type strain. Furthermore, the observation of a small number of free *bipD* mutant bacteria at 6 h p.i. suggests that the *bipD* mutant can escape the phagosome at late time points, a phenotype observed in another TTSS3-defective strain, a *bsaZ* mutant [44]. Consistent with our observations regarding localization of bacteria in single-membrane compartments is that the presence of actin tails and MNGC formation could be observed in RAW 264.7 cells infected with wild-type or *bopA* mutant bacteria as expected because many bacteria are free in the cytosol. Notably, these phenotypes are not observed for *bipD* mutant bacteria as we, and others [9], have shown that bacteria are almost completely sequestered in phagosomes at 6 h p.i..

What then is the specific role of BopA in escape of bacteria from phagosomes? Given that the *bopA* mutant displays a reduced ability to escape the phagosome, it is likely that BopA plays a direct role in disruption of the phagosome membrane, facilitating escape of sequestered bacteria into the host cytosol. Interestingly, it was recently reported that BopA contains a Rho GTPase inactivation domain at its carboxy terminus which may function as a protease or acyltransferase acting on host molecules [45]. Another recent report showed that BopA also contains a cholesterol binding domain [46]. Binding of cholesterol by BopA might lead to the accumulation of cholesterol on phagosome membranes which could limit lysosomal recognition and/or fusion. Such a view is supported by the finding that cholesterol depletion in macrophages infected with *Mycobacterium avium* triggered phagolysosomal or autophagolysosomal formation with consequent bacterial degradation [47]. A detailed investigation of the cellular location and biochemical function of BopA is warranted in order to provide further insight into its role in phagosome escape and intracellular survival. Notably the *bopA* mutant shows reduced virulence in mouse melioidosis models, suggesting that its role in intracellular survival is critical for the pathogensis of *B. pseudomallei*.

### Cytosolic *B. pseudomallei* is resistant to canonical autophagic attack

Wild-type *B. pseudomallei* is highly resistant to LAP and lysosomal killing, as the majority of the intracellular population escapes from phagosomes. Once free in the cytosol, bacteria activate BimA-mediated actin-based motility, replicate, invade adjacent cells via membrane protrusion and form MNGCs (Figure 4). As demonstrated by TEM analysis cytosolic bacteria are highly resistant to uptake by canonical autophagosomes. Indeed, while we were able to observe many autophagosomes in infected RAW 264.7 cells, we identified only one which contained a bacterium. How then do these free bacteria avoid attack by canonical autophagy? It has recently been suggested that it is unlikely that BopA acts in a manner analogous to IcsB of *S. flexneri* which acts to inhibit sequestration of bacteria in autophagosomes by preventing Atg5 binding to BimA [46]. This proposal fits with the results we present here showing that a *bopA* mutant is more susceptible to LAP, but not to cytoplasmic engulfment by autophagosomes.

Autophagosomes engulf several species of cytoplasmic bacteria such as Group A *Streptococcus* and *S. enterica* serovar Typhimurium [17], [48]. Although the signals inducing this autophagic attack are largely unknown, ubiquitination of cytoplasmic bacteria and the binding of the autophagy adaptor protein p62/SQSTM1 play a role in this process (reviewed in [49]). Some bacteria have developed mechanisms to evade or exploit the processes activated by ubiquitination, producing both ubiquitin ligases and deubiquitinases that modulate host defence responses. Recently, the *B. pseudomallei* TssM protein was identified as exhibiting deubiquitinase activity [50], and this activity may represent a mechanism by which *B. pseudomallei* avoids recognition by the machinery of canonical autophagy. Clearly a major challenge that remains is to understand how bacteria free in the cytosol are resistant to attack by canonical autophagy.
